# Female Informants are associated with higher CDR PLUS NACC‐FTLD scores in patients with Frontotemporal Dementia

**DOI:** 10.1002/alz70857_100828

**Published:** 2025-12-24

**Authors:** Juan‐Camilo Vargas‐González, Hessah A Alotibi, Kasey Cortez, Nico Paulo Dimal, Hilary W. Heuer, Leah K. Forsberg, Brian S Appleby, Sami Barmada, Andrea Bozoki, David G. Clark, Yann Cobigo, Ryan Darby, Brad C. Dickerson, Kimiko Domoto‐Reilly, Douglas R. Galasko, Daniel H. Geschwind, Nupur Ghoshal, Neill R. Graff‐Radford, Ian M. Grant, David J Irwin, Ging‐Yuek Robin Hsiung, Lawrence S. Honig, Kejal Kantarci, Gabriel C Leger, Irene Litvan, Ian R MacKenzie, Joseph C. Masdeu, Mario F. Mendez, Chiadi U. Onyike, Belen Pascual, Peter S. Pressman, Eliana Marisa Ramos, Erik D Roberson, Emily J Rogalski, David S. Knopman, Brad F. Boeve, Adam L Boxer, Howard J. Rosen, Carmela Tartaglia, ALLFTD

**Affiliations:** ^1^ Memory Clinic, Toronto Western Hospital, University Health Network, Toronto, ON, Canada; ^2^ University Health Network, Toronto, ON, Canada; ^3^ Memory and Aging Center, UCSF Weill Institute for Neurosciences, San Francisco, CA, USA; ^4^ Mayo Clinic, Rochester, MN, USA; ^5^ Case Western Reserve University School of Medicine, Cleveland, OH, USA; ^6^ University of Michigan, Ann Arbor, MI, USA; ^7^ Michigan Alzheimer's Disease Research Center, Ann Arbor, MI, USA; ^8^ University of North Carolina, Chapel Hill, NC, USA; ^9^ Indiana University School of Medicine, Indianapolis, IN, USA; ^10^ Memory and Aging Center, Department of Neurology, Weill Institute for Neurosciences, University of California, San Francisco, San Francisco, CA, USA; ^11^ Vanderbilt University Medical Center, Nashville, TN, USA; ^12^ Massachusetts General Hospital and Harvard Medical School, Boston, MA, USA; ^13^ University of Washington, Seattle, WA, USA; ^14^ University of California, San Diego, La Jolla, CA, USA; ^15^ University of California, Los Angeles, Los Angeles, CA, USA; ^16^ Washington University School of Medicine, St. Louis, MO, USA; ^17^ Mayo Clinic in Florida, Jacksonville, FL, USA; ^18^ Mesulam Center for Cognitive Neurology and Alzheimer's Disease, Northwestern Feinberg School of Medicine, Chicago, IL, USA; ^19^ Penn FTD Center, Perelman School of Medicine, University of Pennsylvania, Philadelphia, PA, USA; ^20^ University of British Columbia, Vancouver, BC, Canada; ^21^ Columbia University, New York, NY, USA; ^22^ University of California ‐ San Diego, La Jolla, CA, USA; ^23^ University of California, San Diego, San Diego, CA, USA; ^24^ Houston Methodist Research Institute, Houston, TX, USA; ^25^ Johns Hopkins University School of Medicine, Baltimore, MD, USA; ^26^ Layton Healthy Aging and Alzheimer's Disease Research Center, Portland, OR, USA; ^27^ David Geffen School of Medicine, University of California, Los Angeles, Los Angeles, CA, USA; ^28^ University of Alabama at Birmingham, Birmingham, AL, USA; ^29^ University of Chicago, Chicago, IL, USA; ^30^ University of California, San Francisco, San Francisco, CA, USA

## Abstract

**Background:**

Staging disease severity in dementia research requires input from an informant. The Clinical Dementia Rating Sum of Boxes (CDR) is the most used instrument for staging in Alzheimer's Disease (AD); but has been expanded to stage patients with frontotemporal lobar degeneration (FTLD) with the CDR PLUS NACC‐FTLD. An informant effect on the CDR has been reported in AD. The aim of this study is to evaluate whether informant characteristics influence the CDR PLUS NACC‐FTLD in a large cohort of participants with frontotemporal dementia (FTD).

**Method:**

We included participants with a FTLD‐related syndrome from the ALLFTD study. We included participants with a CDR PLUS NACC‐FTLD higher than 0, information on the Montreal Cognitive Assessment (MoCA) scores, and the Neuropsychiatric Inventory Questionnaire (NPI‐Q). We performed a conditional growth model using multilevel linear regression analysis. We performed an exploratory stratified analysis by patient sex.

**Result:**

We included 1411 participants, totalling 2063 visits. The patient's age at the initial visit was 64.7±9.4 years and 44.0% were females. Females were 69.2% of the informants, and the relationship was 79.1% spouse or partner, 10.6% children, 4.1% were siblings and 6.2% other. The CDR PLUS NACC‐FTLD scores were affected by informant characteristics. The CDR PLUS NACC‐FTLD scores were 0.41 (CI 95%:0.08 to 0.75) higher with female informants. The frequency of visits was associated with a CDR PLUS NACC‐FTLD score 0.86 higher (CI 95%:0.17 to 0.56) when visiting at least once a week, 1.46 higher (CI 95%:0.46 to 2.47) when visiting daily, and 0.83 higher (CI 95%:0.06 to 1.60) when living with the patient compared with visiting less than once a week. As expected, CDR‐FLTD scores increased with lower MoCA and higher NPI‐Q scores. In the stratified analysis by sex, we found that female informant was associated with higher CDR PLUS NACC‐FTLD scores only when patients were male.

**Conclusion:**

We found that the CDR PLUS NACC‐FTLD is influenced by informant sex and frequency of visits in patients with FTLD‐related syndromes. These results are similar to those observed in AD patients and underscore the need to recognize that informant characteristics may impact dementia severity scales.

